# Leisure Sports Participants’ Engagement in Preventive Health Behaviors and Their Experience of Constraints on Performing Leisure Activities During the COVID-19 Pandemic

**DOI:** 10.3389/fpsyg.2020.589708

**Published:** 2020-12-09

**Authors:** Young-Jae Kim, Jeong-Hyung Cho, Yeon-Ji Park

**Affiliations:** ^1^Department of Physical Education, Chung-Ang University, Seoul, South Korea; ^2^Da Vinci College of General Education, Chung-Ang University, Seoul, South Korea

**Keywords:** COVID-19, leisure sports, preventive health behavior, leisure constraints, physical activity

## Abstract

This study assessed the demographic characteristics of Koreans engaged in leisure sports activities during the COVID-19 pandemic and the differences in their preventive health behaviors and constraints on leisure activities. For this study, the demographic characteristics (gender, age, marital status, level of participation in leisure sports, years of participation, companions with whom individuals participating in these sports, type of space used for performing the sports, occupation, and average monthly income) of 544 leisure sport participants (men: 46.0%, women: 54.0%; average age: 36.8 and 33.5 years, respectively), who were recruited on a nationwide basis, were examined through an online survey. Then, comparisons between groups were performed using independent *t*-tests, one-way analysis of variance, and multivariate analysis of variance. Women who participated in both indoor and outdoor leisure sports showed higher adoption of health prevention behaviors than their male counterparts, and married individuals who participated in indoor leisure sports showed higher adoption of health prevention behaviors than unmarried participants. Moreover, individuals who participated in both indoor and outdoor leisure sports by themselves had many interpersonal constraints overall, and the group of married individuals who participated in indoor leisure sports showed structural constraints. In conclusion, leisure sports participants have adopted many health prevention behaviors during the COVID-19 pandemic, but this had led to some interpersonal constraints. These results indicate that, in the case of future pandemics, personal and institutional efforts will need to be made to promote participation in leisure sports and prevent excessive social isolation.

## Introduction

The measures implemented to control the COVID-19 pandemic have resulted in people around the world becoming accustomed to new realities such as social distancing (Grenita et al., 2020). However, while social distancing measures can help to reduce COVID-19 infection rates, these measures have also been found to negatively impact physical and mental health by restricting participation in daily activities, travel, leisure activities, physical activities, and various forms of exercise (e.g., as a result of the closure of gyms and restrictions regarding group meetings; Figueroa and Aguilera, 2020; Hossain et al., 2020).

In fact, social distancing-induced reductions in physical activity have been observed in many countries around the world (Ammar et al., 2020; Güzel et al., 2020), and studies have reported that the prolonged nature of the COVID-19 pandemic and the associated mitigation efforts could lead to secondary negative effects concerning the health and welfare of both patients as well as the general population (Gasmi et al., 2020; Lippi et al., 2020).

To reduce their risk of infection, people have adopted preventive health behaviors. Preventive health behaviors refer to activities such as refraining from visiting crowded places, maintaining high levels of personal hygiene (such as avoiding touching one’s eyes, nose, and lips with unwashed hands), and remaining up-to-date on associated events by frequently watching COVID-19-related broadcast programs (Kim and Cho, 2020). Recently, some researchers have suggested that, along with everyday preventive measures, regular physical activity is also needed to prevent the disease (Chen et al., 2020b). Considering this finding, and the fact that low physical activity can increase global mortality (Kohl et al., 2012), it seems clear that public health officials should emphasize the importance of engaging in physical activity in order to maintain good health during the pandemic.

However, in Korea, the COVID-19 situation has restricted people’s ability to participate in leisure sports by implementing social distancing and social isolation measures at the national level (Bond et al., 2020).

In Korea, many public spaces where people can exercise and improve their health, such as public exercise facilities, gymnasiums, and other physical activity-related institutions/facilities, are currently closed (Shin, 2020a), resulting in structural constraints on sports activities. Furthermore, a variety of interpersonal constraints have also arisen as a result of the COVID-19 prevention measures, as restrictions on public gatherings mean that people are unable to meet with friends, peers, groups, or others to participate in leisure activities (Freire, 2020).

Social constraints imposed due to the COVID-19 pandemic limit an individual’s participation in leisure activities and have a huge impact on the external environment and psychological state of the individual (Crawford and Godbey, 1987). Restrictions on participation in various leisure sports can lead to emotional unease including stress, frustration, despair, and conflict. In addition, negative experiences caused by restrictions on leisure activities can lead to several problems in the long run (Rushing et al., 2019).

In light of this, people are engaging in various means of overcoming restrictions for leisure activities during the COVID-19 pandemic. Currently, indoor leisure-sports activities such as “Corona Home Training” have grown increasingly popular around the world, as these activities can be performed alone at home (Lee, 2020). In addition, there are reports of large numbers of people participating in outdoor leisure sports activities such as hiking, which have a relatively lower risk of infection when compared to indoor exercises (Shin, 2020b).

To date, there have been several studies on COVID-19-related preventive behaviors (e.g., Chan et al., 2020), the relationship between preventive measures and regular exercise (Chen et al., 2020b), and limitations on performing recreational activities during quarantine (Güzel et al., 2020). Most of these studies have focused on quarantine environments (López-Bueno et al., 2020) and on making recommendations for improving one’s health by engaging in physical activities while taking care to prevent infection (Nyenhuis et al., 2020). However, there is a lack of research regarding the preventive health behaviors adopted by participants in indoor and outdoor leisure sports activities, as well as the level of constraints these people experience with regard to performing such activities during the COVID-19 pandemic.

Therefore, the purpose of this study was to identify the types of leisure sports people are engaging in during the current pandemic situation, and to analyze, in terms of demographic characteristics, the relationship between participants’ adoption of preventive health behaviors and their constraints regarding performing sports activities. This investigation helps to provide a basic understanding of leisure sports participation and related constraints, which could prove useful in the case of any future pandemics.

## Materials and Methods

### Participants

In order to identify individuals participating in leisure sports activities during the COVID-19 pandemic, we consulted the 2019 Survey on National Leisure Activity, selecting the population comprising Koreans aged between 19 and 65 years (Ministry of Culture Sports and Tourism, 2019). The research targets included people who continuously participated in leisure activities more than once a week. The data collection was conducted online through Embrain Research Company due to limitations in conducting face-to-face surveys in the current situation. Leisure sports types were classified as defined by the Ministry of Culture, Sports, and Tourism, a Korean government agency. The concept of sports as “institutionalized competitive activity using the body” (Coakley, 1986; Lim, 1994), was employed to identify competitive sports events. Participants were asked in advance whether they participated in leisure sports activities, and the sample only included those who reported participating in such activities and who provided written informed consent for the survey. Data were collected online from July 10, 2020 to July 17, 2020; fewer than 50 confirmed COVID-19 cases were reported per day in that week. Overall, a total of 544 individuals participated in this study; their demographic characteristics are presented in Table 1.

**TABLE 1:** Participants’ characteristics, adoption of preventive behaviors, and leisure constraints.

		N	%	Adoption of COVID-19 Preventive Behaviors Mean (SD)	Leisure Constraints Mean (SD)
Gender	Male	250	46.2	3.746 (0.408)	3.396 (0.632)
	Female	291	53.8	3.882 (0.391)	3.408 (0.614)
Age (years)	19–29	199	36.8	3.780 (0.409)	3.415 (0.673)
	30–39	184	34.0	3.782 (0.410)	3.428 (0.604)
	40–49	100	18.5	3.871 (0.396)	3.328 (0.607)
	50–65	58	10.7	3.916 (0.369)	3.406 (0.517)
Marital status	Single	299	55.3	3.786 (0.413)	3.378 (0.624)
	Married	242	44.8	3.860 (0.391)	3.433 (0.620)
Type of leisure sports participated in	Outdoor sports	205	37.9	3.800 (0.399)	3.331 (0.633)
	Indoor sports	336	62.1	3.833 (0.408)	3.446 (0.612)
Experience in performing leisure sports	After the COVID-19 outbreak (After January 2020)	76	14.0	3.747 (0.403)	3.312 (0.653)
	1–2 years	175	32.3	3.821 (0.427)	3.343 (0.600)
	3–4 years	107	19.8	3.853 (0.393)	3.440 (0.582)
	More than five years	183	33.8	3.827 (0.389)	3.360 (0.648)
Companions when performing leisure sports	Alone	245	45.3	3.814 (0.418)	3.483 (0.619)
	Family	104	19.2	3.880 (0.364)	3.381 (0.610)
	Friends of the same or different gender (including colleagues)	192	35.4	3.793 (0.406)	3.312 (0.622)
Type of space used to perform leisure sports activities	Home	215	39.7	3.868 (0.389)	3.430 (0.613)
	Work	45	8.3	3.787 (0.423)	3.472 (0.507)
	Clubs	150	27.7	3.776 (0.395)	3.389 (0.629)
	Other	131	24.2	3.798 (0.429)	3.348 (0.665)
Occupation	Manager	163	30.1	3.876 (0.377)	3.456 (0.649)
	Professional	159	29.4	3.742 (0.420)	3.369 (0.558)
	Service worker	92	17.0	3.892 (0.367)	3.331 (0.630)
	Skilled worker or related person	64	11.7	3.806 (0.403)	3.473 (0.650)
	University student (graduate school student)	63	11.6	3.773 (0.455)	3.381 (0.665)
Average monthly income	= $ 850	59	10.9	3.755 (0.456)	3.300 (0.683)
	$ 851 – 1,700	69	12.8	3.844 (0.381)	3.394 (0.651)
	$ 1701 – 3,420	272	50.3	3.800 (0.399)	3.412 (0.598)
	$ 3,421 – 5,130	94	17.4	3.835 (0.399)	3.465 (0.620)
	= $ 5,130	47	8.7	3.940 (0.399)	3.365 (0.645)
Total		541	100.0		

### Instruments

#### Demographics

The participants provided information concerning their gender, age, marital status, occupation, average monthly income, type of leisure sports in which they participated, years of participation, the people with whom they participated, and their level of participation in these activities.

##### COVID-19 preventive health behaviors

The COVID-19 Preventive Health Behaviors Scale, developed by Kim and Cho (2020), was used in this study. Kim and Cho (2020) created this scale by modifying a scale originally developed by Choi et al. (2016) for use during the Middle East respiratory syndrome epidemic, which referenced the basic guidelines for preventive health behaviors published by the Korean Center for Disease Control and Prevention (Korean Centers for Disease Control and Prevention, 2020). The Preventive Health Behaviors Scale used by Kim and Cho (2020) contains 11 items, all of which are measured using a five-point Likert scale (5 = “strongly agree,” 1 = “strongly disagree”). Higher scores indicate stronger adoption of the preventive health behaviors. Examples of the items are: “I refrain from visiting crowded places,” “I wear masks when I have respiratory symptoms such as fever and cough,” and “I ventilate rooms frequently to ensure that the air indoors is clean.” In Kim and Cho (2020), the internal consistency coefficient of the scale returned a Cronbach’s α of 0.838. The Cronbach’s α for the present study was 0.793, indicating high reliability.

##### Negotiation of leisure constraints

Leisure constraints comprise a variety of factors that limit participation in leisure activities. These include personal constraints related to individuals’ psychological state and personality characteristics, interpersonal constraints manifested through interaction or relationships with others, and structural constraints, which refer to constraints other than personal and interpersonal constraints that may be present in participation in leisure activities. This study used the Leisure Constraints Scale developed by Crawford et al. (1991), as well as the scale used in Cho and Kim (2019). To date, leisure constraints have referred to factors that indirectly and negatively influence leisure participation, hindering individual leisure preferences and undermining the enjoyment obtained from participation in leisure activities (Kay and Jackson, 1991). As such, Cho and Kim (2019) studied factors hindering leisure participation among Korean professionals. The Leisure Constraints Scale comprises 21 items that relate to constraints that can be found in daily life, which are divided into three sub-factors: personal, interpersonal, and structural constraints. Through a meeting with a professor of leisure studies, a doctor of sport sociology, and a doctor of leisure studies, the present authors modified the scale to better adapt it to the COVID-19 situation. For example, in terms of personal constraints, the scale statement “I will not engage in leisure activities that I feel uncomfortable with” was changed to “I feel uncomfortable participating in leisure activities during the COVID-19 pandemic,” and the interpersonal constraint-related item “I do not have friends or partners with whom I can participate in leisure activities” was changed to “I do not have friends or partners with whom I can participate in leisure activities during the COVID-19 pandemic.” The structural constraint-related item “I lack information on leisure activities” was also changed to “There is a lack of information regarding the leisure sports activities in which I can participate during the COVID-19 pandemic.” These reflect the overall changes made to the scale to befit the COVID-19 situation. The scale comprised a total of 15 items, with 5 items each for personal, interpersonal, and structural constraints.

After modification, 5 items were deleted as, according to exploratory factor analysis, their loading values were below 0.06. Consequently, the final scale comprised a total of three sub-factors: three items concerning personal constraints (α = 0.791), three items concerning interpersonal constraints (α = 0.862), and four items concerning structural constraints (α = 0.768), The overall reliability coefficient, measured using Cronbach’s α, was 0.841, showing high reliability. A Kaiser–Meyer–Olkin value of 0.836 was obtained, and the total variance explained was 69.52% (Table 2).

**TABLE 2:** Results of the exploratory factor analysis of the Leisure Constraints Scale modified to reflect the COVID-19 pandemic situation.

Items	Factor 1	Factor 2	Factor 3
**Structural constraints**			
During the COVID-19 pandemic, costs to participate in leisure sports are high.	0.808	0.150	0.039
During the COVID-19 pandemic, there is insufficient time to participate in leisure sports activities.	0.771	0.100	0.166
As a result of the COVID-19 pandemic, it is difficult to obtain the necessary equipment to perform leisure sport activities.	0.750	0.025	0.296
There is a lack of information regarding the leisure sports activities in which I can participate during the COVID-19 pandemic.	0.696	0.124	0.210
**Personal constraints**			
I am uncomfortable about participating in leisure sports during the COVID-19 pandemic.	0.094	0.883	0.161
It is inconvenient to participate in leisure sports during the COVID-19 pandemic.	0.094	0.883	0.233
During the COVID-19 pandemic, I have restricted my participation in leisure activities as a result of the opinions of others.	0.154	0.841	0.107
**Interpersonal constraints**			
People in my social circle have no intention of participating in leisure sports during the COVID-19 pandemic.	0.204	0.126	0.815
I do not have friends or partners with whom I can participate in leisure activities during the COVID-19 pandemic.	0.283	0.138	0.808
My friends (or family) are reluctant to participate in leisure sports because they are worried about becoming infected with COVID-19.	0.133	0.421	0.660
Cronbach’s α	0.791	0.862	0.768
Eigenvalue	2.477	2.470	2.006
Variance (%)	24.766	24.705	20.058
Cumulative variance (%)	24.766	49.471	69.529
KMO = 0.836, χ^2^ = 2202.701, df = 45, *p* < 0.001			

*KMO, Kaiser–Meyer–Olkin value.*

#### Types of Leisure Sports

The types of leisure sports activities in which the participants engaged were assessed using the “Korean Leisure Activity Survey” developed by the Ministry of Culture, Sports and Tourism (Ministry of Culture Sports and Tourism, 2019). Specifically, this survey measured the proportion of the free time that participants dedicated to performing leisure sports, the types of sports they engaged in, and the amount of time they devoted to essential physiological activities (sleeping and eating). The types of leisure sports that one can participate in during the COVID-19 situation were classified into ball games, racquet sports, winter sports, water sports, swimming, jogging, and dance-related sports; for the purpose of the present study, these sports were classified into indoor and outdoor leisure sports. Specifically, indoor leisure sports activities mainly refer to sports activities that can be performed indoors, such as physical training, badminton, table tennis, billiards, bowling, and swimming; outdoor sports activities include soccer, baseball, golf, running (jogging), cycling, and hiking.

### Data Analysis

To analyze the data, first, coding and data-cleaning were performed. All analyses in this study were conducted using SPSS version 25.0. Frequency analysis and descriptive analysis were conducted to examine demographic variables, and exploratory factor analysis was conducted using varimax rotation to resolve problems caused by multicollinearity. In addition, Cronbach’s α tests were performed to ensure the reliability of the measurement scales. Finally, an independent *t*-test and a one-way analysis of variance were used to derive the research results, and multivariate analysis of variance was conducted to simultaneously compare the means for multiple dependent variables (Finch, 2016).

### Procedure

This study was approved by the Institutional Review Board of Chung-Ang University. Data collection was conducted online due to limitations in carrying out face-to-face surveys during the current pandemic situation, which made it challenging to select target participants. Therefore, data were collected with the help of screening questions to choose only those participants who participated in leisure sports during the pandemic. The study was conducted after obtaining informed consent from the participants to complete the survey.

## Results

### Participants’ Characteristics

Table 1 shows the participants’ characteristics. The sample comprised 291 women (53.80%) and 250 men (46.2%), and women were found to show higher levels of adoption of preventive behaviors (*M* = 3.88) and higher levels of leisure constraints (*M* = 3.40). The most common age groups in the sample were 19–29 years (36.8%) and 30–39 years (34.0%), and groups with strong adoption of COVID-19 preventive behaviors were the groups aged 50–65 years (*M* = 3.91) and 40–49 years (*M* = 3.87). However, while the 50–65-years group (*M* = 3.40) had high levels of leisure constraints, the 40–49 years group had the lowest level of leisure constraints (*M* = 3.32). Overall, 299 participants were unmarried (55.3%); married participants showed greater adoption of preventive behaviors (*M* = 3.78) and higher levels of leisure constraints (*M* = 3.37). Overall, 336 people (62.1%) reported participating in indoor leisure sports such as home training, physical training, badminton, table tennis, and billiards during the COVID-19 pandemic, and 205 reported mainly participating in outdoor leisure-sports activities, such as hiking, fishing, soccer, and golf. Typically, those who participated in indoor sports had higher adoption of preventive behaviors (*M* = 3.80) and higher levels of leisure constraints (*M* = 3.33). Additionally, the group who participated in leisure-sports activities with friends and colleagues had lower adoption of preventive behaviors (*M* = 3.83) and lower levels of leisure constraints (*M* = 3.44). Last, service industry workers showed high adoption of preventive behaviors (*M* = 3.89), but the lowest level of leisure constraints (*M* = 3.33).

### Analysis of Differences Among the Participants Regarding Adoption of Preventive Health Behaviors During the COVID-19 Pandemic

Table 3 shows the differences among the participants with regard to adoption of preventive health behaviors during the COVID-19 pandemic (Table 3). For both the participants in outdoor sports activities (*M* = 3.89, *SD* = 0.38) and the participants in indoor sports activities (*M* = 3.87, *SD* = 0.39), women tended to have higher adoption of preventive health behaviors than men. Furthermore, the married participants in the indoor sports activity group (*M* = 3.81, *SD* = 0.38) had higher adoption of preventive health behaviors when compared to the single/unmarried participants (*M* = 3.77, *SD* = 0.41).

**TABLE 3:** Differences in adoption of preventive health behaviors in terms of demographic characteristics.

Variables	Outdoor sports activities (*N* = 205)					Indoor sports activities (*N* = 336)				
		
Health prevention behaviors (*N* = 541)	N	M	SD	F	P	N	M	SD	F	P
**Gender**										
Male	135	3.75	0.39	6.30	0.01	115	3.74	0.42	8.36	0.00
Female	70	3.89	0.38			221	3.87	0.39		
**Age (years)**										
19–29	55	3.81	0.42	2.37	0.071	144	3.79	0.40	1.19	0.31
30–39	72	3.71	0.38			112	3.82	0.42		
40–49	52	3.84	0.37			48	3.89	0.42		
50–65	26	3.92	0.40			32	3.90	0.34		
**Marital status**										
Single	96	3.77	0.41	0.52	0.46	203	3.79	0.41	5.24	0.02
Married	109	3.81	0.38			133	3.89	0.39		
**Experience of participating in leisure sports**										
After the COVID-19 outbreak (after January 2020)	27	3.66	0.38	1.29	0.27	49	3.79	0.41	0.41	0.74
1–2 years	54	3.83	0.44			121	3.81	0.41		
3–4 years	40	3.84	0.40			67	3.86	0.38		
More than 5 years	84	3.79	0.36			99	3.85	0.40		
**Companions when performing leisure sports**										
Alone	69	3.77	0.43	0.80	0.44	176	3.82	0.41	0.85	0.42
Family	44	3.86	0.38			60	3.88	0.34		
Colleague	92	3.78	0.37			100	3.80	0.43		

### Analysis of Differences Among the Outdoor Sports Participants Regarding Leisure Constraints During the COVID-19 Pandemic

Among the outdoor leisure-sports participants, differences in the sub-factors of leisure constraints were analyzed in terms of demographic characteristics. As a result, the characteristics of the participants’ companions (or lack thereof) were found to influence interpersonal constraints (Wilks’ lambda = 0.94, *F* = 1.78 (4.13), *P* < 0.05). Specifically, the group of participants who performed sports alone (*M* = 3.47, *SD* = 0.71) had higher interpersonal constraints when compared to the group who performed sports with family members (*M* = 3.25, *SD* = 0.76) or friends of the same gender (*M* = 3.101, *SD* = 0.85; see Table 4).

**TABLE 4:** Differences in the leisure constraints of outdoor leisure sports participants in terms of demographic characteristics (*N* = 205).

Variables	Individual constraints					Interpersonal constraints				Structural constraints				Wilk’s Λ	*F*	Sig.	η^2^	*Post hoc*
			
	*N*	*M*	*SD*	*F*	*P*	*M*	*SD*	*F*	*P*	*M*	*SD*	*F*	*P*					
**Gender**														0.98	1.09	0.35	0.01	
Male	135	3.79	0.76	0.76	0.38	3.30	0.82	0.99	0.31	2.93	0.88	0.52	0.47					
Female	70	3.89	0.73			3.19	0.75			2.83	0.81							
**Age (years)**														0.97	0.57	0.81	0.00	
19–29	55	3.75	0.90	1.04	0.37	3.32	0.95	0.40	0.74	2.91	0.99	0.30	0.82					
30–40	72	3.94	0.72			3.30	0.77			2.95	0.86							
40–50	52	3.73	0.62			3.17	0.75			2.87	0.80							
50–65	26	3.82	0.77			3.23	0.63			2.76	0.68							
**Marital status**														0.99	0.45	0.71	0.00	
Single	96	3.82	0.80	0.00	0.95	3.32	0.86	0.94	0.33	2.95	0.87	0.61	43					
Married	109	3.82	0.71			3.21	0.74			2.85	0.85							
**Experience of participating in leisure sports**														0.95	0.92	0.49	0.01	
After the COVID-19 outbreak (after January 2020)	27	3.74	0.88	0.99	0.39	3.28	0.63	1.51	0.21	3.00	0.82	0.24	0.86					
1–2 years	54	3.97	0.78			3.41	0.80			2.93	0.92							
3–4 years	40	3.80	0.58			3.05	0.87			2.86	0.88							
More than 5 years	84	3.76	0.76			3.26	0.81			2.86	0.83							
**Companions when performing leisure sports**														0.94	1.78	0.10	0.02	a > c
Alone	69	3.83	0.77	0.52	0.59	3.47	0.71	4.13	0.01	2.97	0.87	0.42	0.65					
Family	44	3.91	0.76			3.25	0.76			2.85	0.93							
Colleague	92	3.77	0.74			3.11	0.85			2.86	0.82.							

### Analysis of Differences Among the Indoor Sports Participants Regarding Leisure Constraints During the COVID-19 Pandemic

Regarding indoor-leisure-sports, participants who were married (*M* = 3.10, *SD* = 0.79) showed higher structural constraints when compared to those who were single (*M* = 2.86, *SD* = 0.78; Wilks’ lambda = 0.97, *F* = 3.12 (7.38), *P* < 0.05). Specifically, similar to the participants in outdoor leisure sports, the group of participants who performed sports alone (*M* = 3.57, *SD* = 0.80) had higher interpersonal constraints when compared to the group who performed sports with family members (*M* = 3.27, *SD* = 0.76) or friends of the same or opposite gender (*M* = 3.22, *SD* = 0.71; see Table 5).

**TABLE 5:** Differences in the leisure constraints of indoor leisure sports participants in terms of demographic characteristics (*N* = 336).

Variables	Individual constraints					Interpersonal constraints				Structural constraints				Wilk’s Λ	*F*	Sig.	η^2^	*Post hoc*
			
	*N*	*M*	*SD*	*F*	*P*	*M*	*SD*	*F*	*P*	*M*	*SD*	*F*	*P*					
**Gender**														0.97	2.34	0.07	0.02	
Male	115	3.87	0.77	2.44	0.11	3.46	0.72	0.79	0.38	3.02	0.69	1.39	0.23					
Female	221	4.01	0.75			3.38	0.84			2.92	0.83							
**Age (years)**														0.98	0.55	0.83	0.00	
19–29	144	3.97	0.80	0.39	0.75	3.40	0.85	0.74	0.52	2.96	0.84	0.03	0.99					
30–40	112	3.92	0.76			3.46	0.77			2.93	0.76							
40–50	48	3.95	0.69			3.27	0.74			2.97	0.79							
50–65	32	4.09	0.66			3.47	0.78			2.96	0.65							
Marital status														0.97	3.12	0.02	0.02	
Single	203	3.90	0.81	3.39	0.06	3.38	0.81	0.76	0.38	2.86	0.78	7.38	0.00					
Married	133	4.06	0.67			3.46	0.78			3.10	0.78							
**Experience of participating in leisure sports**														0.97	0.93	0.49	0.00	1 < 3
After the COVID-19 outbreak (after January 2020)	49	3.74	0.93	2.52	0.05	3.27	0.93	0.95	0.41	2.87	0.79	0.58	0.62					
1–2 years	121	4.00	0.72			3.41	0.78			2.99	0.81							
3–4 years	67	4.11	0.59			3.52	0.72			3.02	0.80							
More than 5 years	199	3.93	0.79			3.40	0.80			2.90	0.75							
**Companions when performing leisure sports**														0.942	3.33	0.00	0.03	a>b,c
Alone	176	3.97	0.82	0.04	0.95	3.57	0.80	7.21	0.00	2.96	0.84	0.07	0.93					
Family	60	3.98	0.75			3.27	0.76			2.97	0.76							
Colleague	101	3.95	0.66			3.22	0.71			2.93	0.71							

## Discussion

This study analyzed, in terms of demographic characteristics, the relationship between preventive health behaviors and leisure constraints among leisure sports participants during the COVID-19 pandemic.

We found that women who participated in both indoor and outdoor leisure sports showed a higher level of adoption of preventive health behaviors as compared to men. This accords with the findings of Kim and Cho (2020), who reported that women had higher adoption of COVID-19-preventive behaviors and better psychosocial health when compared to men. While the present study did not consider the influence of gender on COVID-19 preventive behaviors (Smith, 2019), one reason why women show higher adoption of preventive health behaviors may be that they commonly play the roles of caregivers and front-line health care staff for their families (Wenham et al., 2020). Preventive health behaviors were highly prevalent in the groups aged 50-62 years. A possible reason for this may be that COVID-19 has the highest mortality rate among older people. Thus, they may feel a responsibility to be more mindful of their actions.

Furthermore, COVID-19 is an infectious disease that can be transmitted through contact with other people. All of the participants in outdoor sports activities who avoided enclosed spaces for health-related reasons, and participants of indoor sports such as badminton or swimming, in which there is a risk of infection through contact with the bodily fluids of others, were aware of the risk of infection, and engaged in preventive behaviors. In other words, the fact that there were no differences in terms of gender, age, participation experience, or participants’ companions in relation to the preventive health behaviors for both indoor and outdoor leisure sports participants indicates a generally high level of adoption of preventive health behaviors in the society during the current pandemic. In Kim and Cho (2020), adoption of COVID-19 preventive behaviors was found to be high among the adolescents and adults in their 60s; however, this study did not research the adolescent age group, indicating a gap that should be addressed in future studies. Nevertheless, we can conclude that, in situations of epidemics or infectious diseases, guidelines are required to help people exercise preventive health behaviors while engaging in healthy leisure sports.

During the COVID-19 pandemic, sports participation in Korea can be divided into indoor sports, such as life sports involving virtual reality (Kim, 2020) and home training that is easily done at home (Lee, 2020), and outdoor sports, such as hiking alone or with a single companion (to facilitate social distancing; Kang and Ban, 2020).

Intrinsic factors have been found to be more important than other factors for determining leisure participation (Ajzen and Driver, 1991). However, in the COVID-19 situation, demographic characteristics create differences in the leisure constraints of the participants in indoor and outdoor sports. Our findings indicated that the group of participants who performed sports alone experienced higher interpersonal constraints when compared to those who participated with friends of the same or opposite gender. This may be because COVID-19-related mitigation measures such as social distancing are widespread, causing people to exercise additional caution regarding interpersonal relationships (Kim and Cho, 2020). In other words, constraints on indoor and outdoor physical activities and limitations regarding access to public spaces may have exacerbated the sense of isolation for those who were isolating as a result of the COVID-19 pandemic (Güzel et al., 2020).

Married participants who performed indoor leisure sports generally showed higher levels of structural constraints. This may be because parents with school-going children typically spend a lot of time at home (Jung, 2020), or face limitations performing leisure activities as a result of responsibilities relating to work, housekeeping, etc. In addition, married individuals often engage in family leisure activities. They were reluctant to engage in indoor activities as poor ventilation indoors may lead to higher chances of being infected by COVID-19, which would increase the health risk for their families. Many studies have reported that, while people want to participate in leisure activities, time constraints are a primary preventive reason (Kim et al., 2015). Notably, availability of time has a strong influence on the presence of structural constraints (Iso-Ahola, 1980). Additionally, most leisure activity participation incurs additional costs. Along with time-related factors, economic constraints may be particularly relevant during the COVID-19 pandemic. This is because COVID-19 has created a crisis for the global economy; the International Monetary Fund has reported that the COVID-19 pandemic may result in a deterioration in the economic activity of women worldwide, creating issues concerning income inequality and work-family balance (Korean Herald, 2020). This phenomenon suggests that, in addition to income inequality, there is the potential that structural issues such as “work-leisure conflicts” and “disease-leisure conflicts” will arise in pandemic situations. Therefore, it is necessary to take such changes in lifestyle into account when promoting participation in leisure sports.

There are some limitations to this study. While the study population was selected from across Korea and comprised actual participants in leisure sports during the COVID-19 pandemic, the sample comprised just 541 subjects; nevertheless, this study may be valuable as preliminary research. Second, the results of this study may differ when compared to situations before and after COVID-19, as this study was conducted while the pandemic was still in progress. Third, because the study was limited to Korea, results may differ depending on the restrictions and containment of COVID-19 in other countries. Fourth, this study did not include those who discontinued sports during the pandemic. A follow-up study is necessary to analyze the influence of psychological factors by comparing those who stopped and those who continued to participate in sports activities during the pandemic period.

## Conclusion

In conclusion, leisure-sports participants were found to show high adoption of preventive health behaviors during the COVID-19 situation, and to experience interpersonal constraints when participating in indoor or outdoor leisure sports alone (i.e., the social isolation measures adopted during the COVID-19 pandemic have restricted opportunities for group participation in leisure sports). These results suggest how people participating in leisure sports should adapt to the situation of the COVID-19 pandemic. Furthermore, personal and institutional efforts should be made to promote participation in leisure sports and better social interaction in any similar epidemic in the future.

## Data Availability Statement

The original contributions presented in the study are included in the article/supplementary material, further inquiries can be directed to the corresponding author/s.

## Ethics Statement

The studies involving participants were reviewed and approved by Ethics Committee of Research University of Chung-Ang (1041078-202007-HRSB-170-01). The participants provided their on-line written informed consent to participate in this study.

## Author Contributions

Y-JK designed the study, while Y-JP conducted the study and analyzed the data. Y-JP interpreted the data, and wrote and revised the manuscript. Y-JK and J-HC revised and improved the quality of the analyses performed, critically revised the draft, and made important contributions. All of the authors read and approved the final version of the manuscript.

## Conflict of Interest

The authors declare that the research was conducted in the absence of any commercial or financial relationships that could be construed as a potential conflict of interest.
